# Global Mental Health: sharing and synthesizing knowledge for sustainable development

**DOI:** 10.1017/gmh.2016.22

**Published:** 2016-09-19

**Authors:** K. O'Donnell, M. Lewis O'Donnell

**Affiliations:** Member Care Associates, Global Integration, Geneva, Switzerland

**Keywords:** Crossing sectors, global integration, global mental health, knowledge synthesis, sustainable development, teaching and learning

## Abstract

Global mental health (GMH) is a growing domain with an increasing capacity to positively impact the world community's efforts for sustainable development and wellbeing. Sharing and synthesizing GMH and multi-sectoral knowledge, the focus of this paper, is an important way to support these global efforts. This paper consolidates some of the most recent and relevant ‘context resources’ [global multi-sector (GMS) materials, emphasizing world reports on major issues] and ‘core resources’ (GMH materials, including newsletters, texts, conferences, training, etc.). In addition to offering a guided index of materials, it presents an orientation framework (global integration) to help make important information as accessible and useful as possible. Mental health colleagues are encouraged to stay current in GMH and global issues, to engage in the emerging agendas for sustainable development and wellbeing, and to intentionally connect and contribute across sectors. Colleagues in all sectors are encouraged to do likewise, and to take advantage of the wealth of shared and synthesized knowledge in the GMH domain, such as the materials featured in this paper.

This is the seventh and most extensive paper to date in which we overview the expanding domain of global mental health (GMH) (O'Donnell, 2016). It is part of our efforts to map GMH developments and resources and then share them in concise and accessible ways (e.g. popularizing and translating science and scholarly research via training, publications, and our GMH-Map website, see reference).

A main part of our work has been to encourage colleagues to collaborate across sectors and to identify new ways to leverage their skills, knowledge, interests, and character strengths (e.g. O'Donnell & Lewis O'Donnell, 2013; Swiss Agency for Development and Cooperation, 2016). Multi-sectoral approaches are increasingly being emphasized in addressing global problems, with one important GMH example being the ‘multi-sectoral approach’ highlighted in the *Mental Health Action Plan 2013–2020* (World Health Organization, 2013*b*).

This paper features a *guided index* of recent materials that illustrate the relevance of GMH and multi-sectoral knowledge for promoting sustainable development and wellbeing. It is designed as an *orientation framework* to facilitate GMH understanding and involvement across sectors through its emphasis on sharing and synthesizing knowledge (O'Donnell & Lewis O'Donnell, 2015*d*). More specifically, we have compiled these materials in view of two crucial, global efforts: (a) The global agenda for sustainable development as embodied in *Transforming Our World: The 2030 Agenda for Sustainable Development* (United Nations, 2015*a*); and (b) the global efforts to strengthen humanitarian assistance as summarized in *One Humanity: Shared Responsibility* (United Nations, 2016). These materials are also compiled to practically support Objective 4 in the *Mental Health Action Plan 2013–2020*, ‘To strengthen information systems, evidence and research for mental health’ and especially in low- and middle-income countries (Lora & Sharan, 2015; Ryan *et al.* 2015).

We have organized the paper in two main sections. The first section, Context Resources – Global Multi-Sector Materials (GMS), features seven representative reports on global issues. The second section, Core Resources – GMH Materials, includes seven representative lists of GMH materials. The paper, with its compilation of recent, relevant resources, is an example of a user-friendly tool, a blend of a guided index and an orientation framework, to help colleagues stay current and collaborate together. It is intended for GMH colleagues at all levels of experience, ranging from students to seasoned professionals, as well as colleagues at all levels of experience in different sectors (e.g. health, development, humanitarian, business, civil society, governments).

## GMH and global integration

We define GMH broadly as an international, interdisciplinary, culturally sensitive, and multi-sectoral domain which promotes human wellbeing, the right to health, and equity in health *for all*. It encourages healthy behaviours and lifestyles; is committed to preventing and treating mental, neurological, and substance use conditions (MNS); and seeks to improve policies and programs, professional practices and research, advocacy and awareness, and social and environmental factors that affect health and wellbeing (O'Donnell, 2012*a*). This GMH definition, in both its breadth and its emphasis on health and wellbeing *for all*, points toward the many interactive areas that GMH colleagues can pursue themselves and with colleagues in other sectors.

We also view GMH through the broader framework of ‘global integration’ (GI) (O**’**Donnell & Lewis O**’**Donnell, 2015*b*, *c*, 2016). GI refers to how people are actively and skillfully integrating their lives with global issues. It involves connecting relationally and contributing relevantly on behalf of human wellbeing and the major issues facing humanity, in light of one's integrity and core values (e.g. ethical, humanitarian, faith-based). We have found this framework to be relevant for the increasing numbers of mental health professionals (MHPs) and colleagues across sectors who want to exchange knowledge and be meaningfully involved in our globalizing world. It also reflects the growing interests and involvements in the rise of citizen participation and global citizenship (United Nations Educational, Scientific, and Cultural Organization, 2014; United Nations, Department of Public Information, 2016).

**Fig. 1.**
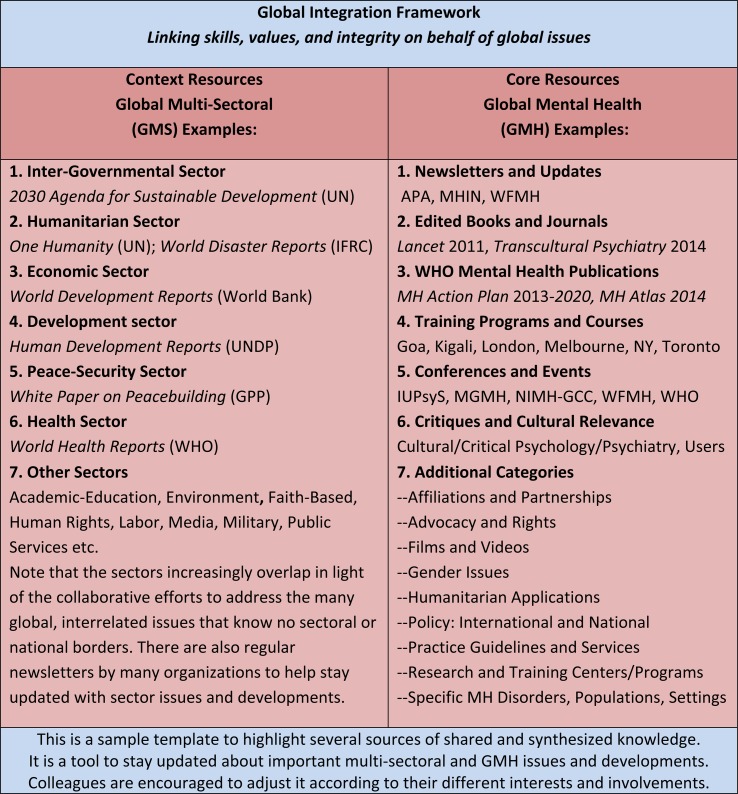
An organizing grid for the article and for GMS and GMH knowledge.

This paper then, with its emphasis on the GMS context and the GMH core, further maps important resources and developments to inform colleagues’ work in wellbeing and sustainable development. We have also organized the main material in the paper in terms of the GI framework, as summarized in Fig. 1.

### Context resources – GMS materials

The seven world reports below, and the additional materials listed in their ‘More resources’ subsections, overview major issues and efforts for development and wellbeing. Colleagues can be both guided and goaded by these materials as they reflect the unfolding reality that ‘the context is crucial for the core’ – that is, understanding the global context is essential for working effectively in one's core emphasis, be it a discipline, organization, sector, country, specific issue, or domain like GMH. We note that both the reports and the materials listed in this section, many of which are available online in multiple languages, are just a sampling of course, among the hundreds of excellent items that are available.

We encourage you to read the overviews/summaries and more if possible as well as to identify the main newsletters-updates from each sector that interest you. We have gone over many of these multi-sectoral reports repeatedly, and often along with several colleagues, in order to consider their serious implications for our world, our work, and our personal lives. One example of how we interact with colleagues from different sectors is the Trio Gatherings (see reference) that we periodically convene in our home. These informal gatherings provide an alternative and relaxed place (outside of Geneva offices) to discuss global issues for mutual learning and mutual support. In GI terms, the gatherings are an additional way to connect relationally and to contribute relevantly with colleagues.

1. *Transforming Our World: The 2030 Agenda for Sustainable Development* (United Nations, 2015*a*). This *Agenda was* developed over the last few years with extensive input/debate from governments and civil society. It is a global plan of action that endeavors to be both aspirational and achievable as it focuses on the overlapping areas of People, Planet, Prosperity, Peace, and Partnerships.

*More resources:* (a) You can track progress on the SDGs via the UN Sustainable Development Knowledge Platform (see reference). Watch the 2-min inspirational video by the UN on the Sustainable Development Goals (see reference), the UN Year in Review video reports (10–15 min, reference), and the videos of the speeches (see reference) made by world leaders at the UN General Assembly following the adoption of the sustainable development agenda, on the UN WebTV website. Also helpful are the selected Online Resources related to SDGs from UN agencies (22 October 2015, United Nations Regional Information Centre, reference). See also (b) the resources on the website for the United Nations Environment Programme (2012), which includes the *Global Environment Outlook 5: Environment for the Future We Want* (2012) and information on *GEO6* to be published in 2017; as well as *Laudato Sí: On Care for our Common Home* (2015), the environmental encyclical by Pope Francis (2015) and the Paris Climate Change Conference and the *Paris Agreement* (United Nations Framework Convention on Climate Change, 2016); (c) the #FundamentalSDG website (see reference), a coalition of organizations working to include mental health more fully into the SDGs (currently focusing on the global indicators which are to be finalized in the first half of 2016); (d) United Nations Academic Impact (see reference), uniting academic institutions in training, research, and action for the SDGs; and (e) the Gyeongju Action Plan: Education for Global Citizenship (United Nations, Department of Public Information/NGOs, 2016).

1. *One Humanity: Shared Responsibility* (United Nations, 2016). This document is the report of the UN Secretary-General for the World Humanitarian Summit (WHS). The report, via its *Agenda for Humanity*, calls upon the world community to rally around five core responsibilities on behalf of the millions of people affected by humanitarian crises in our world. ‘1. *Political leadership* to *prevent and end conflicts*….2. *Uphold the norms that safeguard humanity*….3. *Leave no one behind*….4. *Change people's lives: from delivering aid to ending need*…5. *Invest in humanity*. (pp. 48–60)

*More resources*: (a) You can track the WHS progress on the WPS website (see reference) as well as access key documents such as *Restoring Humanity: Global Voices Calling for Action* (United Nations, 2015*b*), the synthesis report derived from the various regional and thematic consultations 2014–2015 en route to the WHS. (b) Other WHS resources include the photo overview of *One Humanity*; the *Co-Chairs’ Summary* of the WHS Global Consultation held in Geneva 14–16 October 2015 (World Humanitarian Summit, 2015*a*), the video recordings of the Global Consultation plenary sessions (World Humanitarian Summit, 2015*b*), and the archived videos of several WHS sessions. (c) See also the *Humanitarian Accountability Report: On the Road to Istanbul* (Core Humanitarian Standard Alliance, 2015) and the advocacy paper on mental health and psychosocial support prepared for the WHS (United Nations Inter-agency Standing Committee Reference Group on MHPSS in Humanitarian Emergencies, 2015).

1. *World Disaster Report 2015: Local Actors* – *The Key to Humanitarian Effectiveness* (International Federation of Red Cross and Red Crescent Societies, 2015). This Report calls for a shift to the ‘localization’ of aid. It addresses the often overlooked and often central role of local actors in helping to deal with crisis as well as the dearth of humanitarian funding that such actors and their local organizations receive.

*More resources*: (a) *See the World Disasters Report: Focus on Culture and Risk* (International Federation of the Red Cross and Red Crescent Societies, 2014), the *World Humanitarian Data and Trends 2015* (UN Office for the Coordination of Humanitarian Affairs, OCHA 2015), *Leaving No One Behind: Humanitarian Effectiveness in the Age of the Sustainable Development Goals* (OCHA, 2016), and *Humanitarian Action for Children 2016* (United Nations Children's Emergency Fund, 2016); (b) the newsletter-updates from the UN Inter-Agency Standing Committee (IASC, reference), the Overseas Development Institute (see reference), UN-OCHA's reliefnet (reference), ACAPS (e.g. global emergency overview, see reference), and the Active Learning Network for Accountability and Performance (ALNAP, see reference); (c) the news and analyses from IRIN (see reference); webinars by Professionals in Humanitarian Assistance and Protection (PHAP, see reference); (d) global/regional/national reports on the refugee crises and other issues, such as the inter-agency *Regional Refugee and Migrant Response Plan for Europe* (United Nations High Commissioner for Refugees and International Organization for Migration, 2016) and *WFP in Iraq: 2015 in Review* (World Food Program, 2016).

1. *World Development Report: Mind, Society, and Behavior* (World Bank, 2015*b*). This is the first time the World Bank's annual development report has focused extensively on the application of the behavioral sciences to development. The Report aims to integrate recent findings on the psychological and social underpinnings of behavior to make them available for more systematic use by both researchers and practitioners in development communities.’ (p. 2)

*More resources*: (a) Watch the lecture-interview of Dr Jeffrey Sachs (2015) on *The Age of Sustainable Development* as well as his Introduction to Sustainable Development course (Corsera, 2016). See also: (b) the latest wellbeing-related reports, the *State of Global Well-Being* (Gallop-Healthways, 2014); *How's Life? 2015: Measuring Well-Being* (Organization for Economic Cooperation and Development, 2015); and the *World Happiness Report Update 2016* (Sustainable Development Solutions Network, 2016); and (c) the statement from the inter-faith consultation that was convened by the World Bank, *Ending Extreme Poverty: A Moral and Spiritual Imperative* (World Bank, 2015*a*) and the *Corruption Perception Index 2015* (Transparency International, 2016). We note that the terms ‘moral imperative,’ ‘moral responsibility,’ etc. are often used when referring to the UN agenda for sustainable development. Even as there is ‘no health without mental health’ so also, in our view, ‘there is no health-development without moral health-development’ (O'Donnell and Lewis ODonnell, 2015*a*).

1. *Human Development Report: Sustaining Human Development–Reducing Vulnerabilities and Building Resilience* [United Nations Development Program (UNDP), 2014]. This Report looks at the steady albeit unevenly distributed rise of human development around the world. Development progress is laced with a sense of ‘precariousness’ due to the undermining realities of crime, corruption, disasters, war, discrimination, etc., all which add to individual and community vulnerability.

*More resources*: (a) See the *Human Development Report 2015: Rethinking Work for Human Development* (UNDP, 2015) and the online interactive version (see reference) as well as the upcoming 2016 report themed *The Way Ahead* (25th edition); (b) the various and ongoing UNDP reports at the national and regional levels, such as the *Arab Human Development Reports*; and (c) as a further example of regional development issues and analyses, *Arab Human Development in the 21st Century: The Primacy of Empowerment* (Korany, 2014).

1. *White Paper on Peacebuilding* (Geneva Peacebuilding Platform, 2014). This succinct Report is based on a multi-stakeholder initiative to explore peacebuilding practice. It is organized into three sections which summarize perspectives on the challenges, opportunities, and future of peacebuilding practice.

*More resources*: (a) See the global reports: *Positive Peace Report 2015* (Institute for Economics and Peace, 2015); *Global Risks 2015* (World Economic Forum, 2015); *Global Burden of Armed Violence 2015: Every Body Counts* (Geneva Declaration on Armed Violence and Development, 2015); the annual *Global Reports* from the United Nations High Commissioner for Refugees (see reference); *World Report 2015* (Human Rights Watch, 2015); *Crisis Overview 2015: Humanitarian Trends and Risks 2016* (ACAPS, 2016); and Risk Management Toolkit in Relation to Counterterrorism Measures (Norwegian Refugee Council, 2015). See also: (b) Peace and Collaborative Development Network's website (see reference), with daily updates/resources shared by the networks members, and the website of The Carter Center (peace, health, human rights see reference); the special issue on Peace Psychology in the *American Psychologist* (American Psychological Association, 2013); and (c) the ICRC's *International Review of the Red Cross* (recent issues of this journal focus on Sexual Violence in Armed Conflict (Summer, 2014) and Violence Against Health Care (Spring, 2013; Summer, 2013).

1. *Disease Control Priorities* (3rd ed). (University of Washington, Department of Global Health and Institute for Health Metrics and Evaluation, 2015–2016). This third edition is organized into nine topical volumes, with volume four being *Mental, Neurological, and Substance Use Disorders* (2015). Overall this new edition endeavors to provide up to date information on the burden of global disease, including intervention and program effectiveness.

*More resources*: (a) See Global Health 2035: A World Converging within a Generation, The Lancet Commission on Investing in Health reference; (b) *World Health Report 2013: Research for Universal Health Coverage*, (World Health Organization, 2013*d*), and *World Health Statistics 2015* (WHO, 2015*d*); (c) two among many examples of organizational coalitions with major advocating roles are the Peoples Health Movement (see reference) and the NCD Alliance (see reference); and (d) three examples of organizations, focusing on training (among the growing numbers of academic institutions/programs) are the Global Health Center at the Graduate Institute Geneva (global health governance/diplomacy emphases see reference), the Global Health Learning Center (online courses, see reference), and the World Federation of Academic Institutions in Global Health (WFAIGH).

### Core resources – GMH materials

This section lists seven categories of recent materials that are shaping the contours and the content of the GMH domain. The seven GMH categories include: newsletters and updates, edited books and special journal issues, World Health Organization publications, training programs and courses, conferences and events, GMH critiques, and GMH categories to be developed. Note that other materials could not be listed due to space limitations many of which are included on GMH-related websites (see item 1 below). We note that one of the great challenges for GMH is to unite further, that is to cooperate,collaborate, and leverage our many ‘voices’ to advocate for GMH at the global policy level and integrate GMH into sectoral, regional, local, and global agendas.

#### GMH newsletters and updates

Here are 10 GMH-related newsletters/bulletins (organized alphabetically by organization).

- Centre for Global Mental Health, London School of Hygiene and Tropical Medicine, and King's Health Partners (monthly, see reference).
- Centre for International Mental Health, University of Melbourne (monthly, see reference).
- Gulbenkian Global Mental Health Platform (monthly, see reference).
- International Union of Psychological Science (monthly, see reference).
- Mental Health Innovation Network (monthly, see reference).
- Movement for Global Mental Health (monthly, see reference).
- National Institute of Mental Health, USA (quarterly, see reference).
- Office of International Affairs, American Psychological Association (4–6 times/year, see reference). See also the biweekly news bulletin emphasizing opportunities for international involvement in mental health.
- World Federation for Mental Health (quarterly, see reference).
- World Health Organization (twice a year, see reference).

In addition to the above items, see these sampling of websites for news and resources:

- in2mentalhealth (including the description/links for 15 GMH websites/online communities, see reference).
- GMH-Map (Member Care Associates; emphasizing orientation materials and overviews, see reference).
- Grand Challenges in Global Mental Health Initiative (National Institute of Mental Health and Global Alliance for Chronic Disease, see reference).
- Mental Health and Psychosocial Support Network (a community of colleagues sharing resources/good practice, with special interest groups and forums, see reference).
- MINDbank (World Health Organization; a major online platform for MH resources–national/international, human rights, development, etc. see reference).
- Psychology Resources Around the World (International Union of Psychological Science see reference).

#### GMH Edited books and special journal issues

There is a steady and growing stream of edited texts and journals related to GMH, crucial for training-practice and providing colleagues from around the world the platforms to share their research, implementation experiences, and perspectives.

*Texts* (all edited compilations of articles, organized by publishing date):

- *Global Mental Health: Trauma and Recovery* (Mollica, 2011).
- Community Mental Health: Putting Policy into Practice Globally (Thornicroft *et al.* 2011).
- *21st Century Global Mental Health* (Sorel, 2012).
- Global Mental Health: Principles and Practice (Patel *et al.* 2013).
- *Public Mental Health: Global Perspectives* (Knifton and Quinn, 2013).
- Improving Mental Health Care: The Global Challenge (Thornicroft *et al.* 2013).
- Proceedings of the 30th International Congress of Psychology (Cooper and Ratele, 2014):
- Psychology Serving Humanity (volume one): Majority World Psychology
- Psychology Serving Humanity (volume two): Western Psychology

- *Global Mental Health Trials* (Thornicroft and Patel, 2014).
- *Essentials of Global Mental Health* (Okpaku, 2014).
- *Global Mental Health: Anthropological Perspectives* (Kohrt and Mendenhall, 2015).
- Re-Visioning Psychiatry: Cultural Phenomenology, Critical Neuroscience, and Global Mental Health (Kirmayer *et al.* 2015).

*Special Journal Issues* (organized by date):

- *The Lancet* (September 2007).
- *PLOS Medicine* (2009–2010)
- *The Lancet* (October 2011)
- *PLOS Medicine* (May 2012)
- *PLOS Medicine* (April–May 2013)
- *International Health* (March 2013)
- *Global Mental Health* (open access journal, since 2014)
- *International Review of Psychiatry* (October 2014)
- *Intervention: Journal of Mental Health, Psychosocial Work, and Counselling in Areas of Armed Conflict* (December 2014)
- *Transcultural Psychiatry* (December 2014)
- *Academic Psychiatry* (several GMH articles published in 2015–2016; use ‘GMH’ in the search engine, see reference).

#### World Health Organization, Mental Health Publications

WHO has an extensive listing of their Mental Health Publications. Here are five recent publications.

- Building Back Better: Sustainable Mental Health Care after Emergencies (2013*a*)
- Mental Health Action Plan: 2013–2020 (2013*b*)
- Preventing Suicide: A Global Imperative (2014b)
- *Mental Health Atlas 2014* (2015*a*)
- mhGAP Humanitarian Intervention Guide (2015*c*)

#### Training programs and courses

There are a growing number of GMH-related courses taught as part of graduate study programs and some which are available online. In addition there are or have been specific GMH overview courses at various universities such as Columbia University (Dr Kathleen Pike), Duke University (Dr Brandon Kohrt), Fordham University (Dr Andrew Rasmussen), George Washington University (Dr Eliot Sorel), and Johns Hopkins University (Dr Judith Bass). Also note the ongoing special GMH seminars and additional courses as part of GMH programs such as those at Columbia University, McGill University (reference), University of Toronto (reference), University of Washington (reference), and Yale University (reference), and at schools of public health and global health. There are a growing number of emphases in psychiatry programs on GMH that include options for training and experience, including fellowships. Psychology programs for the most part are not yet including these options. Boston College has a Global Practice Concentration in its School of Social Work (reference).

These developments above will certainly be mirrored in other areas of the world, and vice versa. They are part of the larger academic and social emphases of international relations/international studies and now areas which include the term ‘global’ such as ‘global affairs.’ Some examples among many are the University of Notre Dame's School of Global Affairs (opening August 2017, see reference) and the many global engagement priorities and opportunities integrated into undergraduate and graduate majors (e.g. University of Chicago (reference); American University's School of International Service (reference); Connecticut College's Center for International Studies and the Liberal Arts (reference)).

### Graduate academic programs

- Chicago School of Professional Psychology, International Psychology, online doctoral studies with concentrations in Organizations/Systems or Trauma Services (see reference).
- King's College London, Department of Psychiatry, Human Services and Population Research Department (HSPR), MSc Global Mental Health (see reference).
- London School of Hygiene and Tropical Medicine and King's College London's Institute of Psychiatry, MSc Global Mental Health (see reference).
- Universidade Nova de Lisboa, Faculdade de Ciências Médicas, International Masters in Mental Health Policies and Services (see reference).
- Global and Cultural Mental Health (University of Melbourne, School of Population and Global Health) offers research degrees, short courses, and international and multicultural mental health leadership programs (see reference).
- University of Glasgow, MSc/Postgraduate Diploma and Postgraduate Certificate in GMH (see reference).
- University of Glasgow, online courses: MSc/Postgraduate Diploma/Postgraduate Certificate (see reference).
- William James College offers a concentration in GMH as a specialization option for any of its academic programs, in association with its Center for Multicultural and Global Mental Health (see reference).

### Courses

- Global and Cultural Mental Health (University of Melbourne, School of Population and Global Health) offers research degrees, short courses, and international and multicultural mental health leadership programs (see reference).
- Global Mental Health: Trauma and Recovery Certificate Program (Harvard Program in Refugee Trauma) combines on-site learning in Italy and web-based learning (see reference).
- International Diploma in Mental Health Law and Human Rights (Indian Law Society and World Health Organization) is a distance learning program to accommodate working professionals across the globe (see reference).
- Leadership in Mental Health Course (Sangath Centre, London School of Hygiene and Tropical Medicine) in Goa, India (see reference).
- Mental Health First Aid (MHFA), with links for training in over 20 countries (e.g. in Asia: Cambodia, Hong Kong, Nepal, Singapore, Thailand, etc., see reference).

### Other training examples from 2015 to 2016 (organized by date).

- Master Class on Implementation Science (Kings College London) (1–2 June 2015, see reference).
- Summer Institute in Mental Health Research (Johns Hopkins University, Bloomberg School of Public Health) online course on Mental Health in Humanitarian Settings (8–26 June 2015, see reference).
- Global Perspectives on Mental Wellbeing–Knowledge Exchange/Summer School, (University of Glasgow and University of Rwanda), Kigali, Rwanda (15–19 June 2015, see reference).
- Centre for Global Mental Health Summer School (King's College London), London (14–17 September 2015, see reference).
- Mental Health in Complex Emergencies (Fordham University, Institute of International Affairs; International Medical Corps; HealthNet TPO), Addis Ababa, Ethiopia (20–30 September 2015, see reference).
- Mental Health and Psychosocial Support in International Humanitarian Settings (Johns Hopkins University, Bloomberg School of Public Health) online course (31 May–17 June 2016, see reference).
- Mental Health Leadership Course for Young African Psychiatrists (Ethiopian Psychiatric Association) Addis Ababa, Ethiopia (June 2016, see reference).
- Global Health and Health Diplomacy (Lisbon Institute of Global Mental Health). Lisbon, Portugal. (June 2016, see reference).
- Global mHealth Research Training Institute (Fogarty International Center) Bethesda, MD, USA (June 2016, see reference).
- Eastern Mediterranean Mental Health Leadership Course (WHO EMRO Office) Cairo, Egypt (July 2016, see reference).
- Summer Institute in Global Mental Health and Psychosocial Support (Teachers College, Columbia University) New York (5–10 July 2016, see reference).
- HSPR Summer School 2016: Global Mental Health – Research and Action (Institute of Psychiatry, Psychology, and Neuroscience, King's College and London School of Hygiene and Tropical Medicine) London (15–18 August 2016, see reference).
- Mental Health in Complex Emergencies (Fordham University, Institute of International Humanitarian Affairs), Geneva, Switzerland (9–19 October 2016, see reference).
- Leadership in Mental Health course (Sangath Centre and the London School of Hygiene and Tropical Medicine) Goa, India (November 2016, see reference).

#### GMH conferences and events (organized by date)

This section lists many of the GMH-related conferences and consultations from 2013 to 2016. These gatherings have helped colleagues share research activities and resources, discuss issues together, as well as form closer relationships (the latter though may not necessarily happen unless interactive and social times are intentionally built into conference programs). Many of the presentations and other materials from these gatherings are available online. Hence, even though one is not able to attend, it is still possible to ‘participate’ and benefit via the materials that are available (e.g. power points/videos of the presentations, papers, and summary reports).

There are many other mental health-related gatherings that overlap with and include GMH topics that could be added to the list below. Some of the organizations providing updated listings are done by the American Psychological Association (International Meetings, see reference) the International Union of Psychological Science (Calendar of Upcoming Conferences, see reference), and Columbia University's Global Mental Health Program (All Upcoming Events, see reference). It would also be helpful to list other major gatherings/events prior to 2013, including links to presentations/materials, starting perhaps with the launch of the mhGAP Program in 2008.

One suggestion for making the most of these gatherings is to meet together periodically with colleagues, intentionally including those from other sectors, to review and discuss some of the materials (and/or viewing any of the growing number of live-streamed webinars). Going one step further: why not set up an informal ‘net-hub’ in your area/setting in order to meet periodically for mutual learning and support, and further connect and contribute to GMH. For a brief proposal on GMH nets/hubs, see GMH-Geneva (O'Donnell and Lewis O'Donnell, 2012).

- March 2013: The World in Denial? GMH Matters (Royal Society of Medicine and Royal College of Psychiatrists, 2013) London.
- May 2013: Advances in Global Mental Health Research and Research Capacity Building (National Institute of Mental Health 2013) USA.
- May 2013: Cultural Psychiatry and Global Mental Health (Free University, 2013) The Netherlands.
- August 2013: Third Global Mental Health Summit, with power points/summary and videos (Movement for Global Mental Health, 2013) Thailand.
- September 2013: GMH Forum, Sustainable Development though Global Action: The Case for Investing in Mental Health, with power points/and papers (Centre for Global Mental Health, 2013) London.
- October 2013: International Forum on Innovation in GMH, with videos and power points (Gulbenkian Platform, 2013) Lisbon.
- October 2013: mhGAP Forum, launch of the *Mental Health Action Plan 2013–2020* (World Health Organization 2013*b*, *c*) Geneva.
- October 2013: World Mental Health Day, Mental Health and Older People (World Federation for Mental Health, 2013).
- October 2013: Hidden Pictures film, screening live and online internationally (Ruston, 2013).
- December 2013: Launch of the MINDbank website (see reference).
- June 2014: Solving the Grand Challenges in GMH (National Institute of Mental Health and Grand Challenges Canada, USA, 2014).
- September 2014: mhGAP Forum and Launch of the *World Suicide Report* (WHO, 2014*a*, *b*), including the Forum Report and two animated videos on depression: I Had a Black Dog (Johnstone, 2012) and Living with a Black Dog (Johnstone, 2014; World Health Organization, 2014*a*) Geneva.
- October 2014: World Mental Health Day, Living with Schizophrenia (World Federation or Mental Health, 2014).
- February 2015: Mental Health and Wellbeing in Children, panel discussion, video (World Innovation Summit for Health, 2015) Qatar.
- April 2015: Culture and Global Mental Health Society for the Study of Psychiatry and Culture, 2015) Providence, RI, USA.
- April 2015: Mental Health for All: Connecting People and Sharing Experience (World Federation for Mental Health, 2015*b*) France.
- April 2015: Psychology Day at the UN: Reducing Health Inequalities Within and Among Countries: Psychology's Contributions to the United Nation's Post-2015 Global Agenda (Psychology Day at the UN, 2015, see reference) New York.
- June 2015: Crossing Boundaries: Meeting the Needs of Refugee Communities Around the Globe, (Center for Multicultural and Global Mental Health, William James College, 2015) USA.
- July 2015: Pan-African Conference on Trauma and Mental Health Across the Lifespan (Peter C. Alderman Foundation, 2015) Nairobi.
- August 2015: Psychological Contributions to Solving Global Problems in the 21st Century (International Council of Psychologists, 2015) Toronto.
- October, 2015: mhGAP Forum, Mental Health Innovations and Their Uptake into Policy and Practice (World Health Organization, 2015*b*) Geneva.
- October 2015: World Mental Health Day, Mental Health and Dignity (World Federation for Mental Health, 2015*a*).
- October 2015: World Congress of the World Federation for Mental Health (World Federation for Mental Health, 2015*c*) Cairo.
- October 2015: Global Challenges and Cultural Psychiatry – Natural Disasters, Conflicts, Insecurity, Migration, and Spirituality (World Association of Cultural Psychiatry, 2015) Mexico.
- November 2015: Fourth Global Mental Health Summit (Movement for Global Mental Health, 2015) India.
- April 2016: Out of the Shadows: Making Mental Health a Global Development Priority (World Bank and World Health Organization) Washington DC (see reference).
- April 2016: Solving the Grand Challenges of Global Mental Health: Maintaining Momentum on the Road to Scale Up (National Institute of Mental Health, USA and Grand Challenges, Canada) Washington DC (see reference).
- April 2016: Psychology Day at the UN: From Vulnerability to Resilience: Using Psychology to Address the Global Migration Crisis (Psychology Day at the UN, 2016) New York.
- July 2016: International Congress of Psychology, Diversity in Harmony: Insights from Psychology, (International Union of Psychological Science) Japan (see reference).

#### GMH critiques and cultural relevance

GMH is not without its critics. Here are some examples, which critique certain emphases and perceived emphases of GMH. The references and reference sections in the materials below provide a fuller listing. From our perspective, there is much to appreciate in these critiques as well as much to challenge. Here are some examples.

- Toward a New Architecture for Global Mental Health (Kirmayer and Pedersen, 2014). This is a helpful review of some of the concerns with GMH–a good place for a ‘one-stop’ overview. Some highlights include recognizing the influences and agendas that are shaping GMH or any ‘global’ area; understanding and prioritizing cultural formulations of distress; ensuring that the ‘humanity’ of the recipients of GMH services is preserved and that the human qualities of empathy and authentic care are not lost as we apply manuals and good practice guides and statistics across peoples/cultures. There is an appreciation that doing GMH well is important yet not easy!
- Patel (2014) identifies four categories of GMH critique, in his article, Why Mental Health Matters to Global Health. The concerns are: ‘(a) that the ‘diagnoses’ of mental disorders are not valid because there are no biological markers for these conditions; (b) that the strong association of social determinants undermines the use of biomedical interventions; (c) that the field is a proxy for the expansion of the pharmaceutical industry; and (d) that the actions of global mental health are equivalent to ‘medical imperialism’ and it is a ‘psychiatric export’ (p. 777).
- Interview in the Movement for Global Mental Health Newsletter (May 2014) with China Mills and her book *Decolonizing Global Mental Health: The Psychiatrization of the Majority World* (2013).
- Global Mental Health and its Discontents (Bemme and D'souza, 2012) is a summary of a conference/workshop at McGill University on GMH: Bridging the Perspectives of Cultural Psychology and Public Health.
- Afterword: Against ‘Global Mental Health (Sommerfield, 2012) is an article summarizing many concerns about GMH from anthropological and cultural psychiatry perspectives.
- For additional perspectives see the International Critical Psychiatry Network and Kohrt and Mendenhall's perspectives from anthropology (2016).

#### GMH – to be developed

Here are some of the many categories of resources within the GMH domain that can be further organized, including by region and language. Note that a ‘resource primer’ with an extensive listing of GMH materials was published in *International Perspectives in Psychology: Research, Practice, Consultation* (O'Donnell, 2012*b*).

- Affiliations and Partnerships – e.g. GMH Partnering (Member Care Associates, see reference).
- Advocacy and Rights – e.g. *QualityRights* Project and Toolkit (WHO, 2012).
- Alcohol and Substance Abuse – e.g. Management of Substance Abuse (World Health Organization, see reference).
- Films, Documentaries, and Videos – e.g. Breaking the Chains (Colucci, 2013), Like a Death Sentence (Human Rights Watch, 2012), Hidden Pictures (Ruston, 2013), Mental Health for All and by All (Patel, 2012).
- Gender Issues – e.g. Gender and Mental Health (French, 2011).
- Humanitarian Applications – e.g. GMH Humanitarian (Member Care Associates, see reference).
- Policy: International and National – e.g. *Mental Health Action Plan 2013–2020* (World Health Organization, 2013*b*).
- Practice Guidelines and Services – e.g. Putting evidence into practice: The PLOS medicine series on global mental health practice. (Patel *el al.* 2012).
- Research and Training Centers/Programs – e.g. Global Mental Health Research Program (National Institute of Mental Health, see reference) and Collaborating Centres (World Health Organization, see reference).
- Voices and Stories – e.g. GMH Voices (Member Care Associates, see reference).
- Webinars – e.g. links to upcoming and archived GMH webinars related to GMH.

### Working together – sector connectors

It is challenging to track with the expanding GMH domain. In fact, it can even be daunting and contribute to the information overload that regularly besets us. But in many ways we welcome this challenge as it is an indication of GMH's steady development and influence. One important follow-up to this paper could be a survey to assess how GMH colleagues (a) identify, stay current with, and utilize contextual and core materials; (b) are involved in different sectors; and (c) make use of the many international news and media sources on offer (e.g. see our GI Update, Staying Updated – Navigating the News; O'Donnell and Lewis O'Donnell, 2015*e*). Another follow-up item would be for some of the current information brokers (e.g. those responsible for GMH-related websites and newsletters) and other GMH colleagues to consult together in order to further develop sustainable, coordinated efforts in sharing news, updates, and resources.

This paper stresses the importance of sharing and synthesizing both core GMH materials and contextual GMS materials (e.g. world reports and key updates). Strategic knowledge sharing and synthesis also supports civil society's increasing, informed involvements in global affairs and, in particular, with the United Nations in the agenda for sustainable development and wellbeing (e.g. Partnerships Engagement for the Sustainable Development Goals, United Nations, see reference). Although not without its flaws and failures (like any human institution), the United Nations continues to be crucial for promoting and protecting the overlapping areas of human security/peace, human rights/dignity, and social progress/better standards of life, as highlighted in Preamble of the *Charter of the United Nations* (United Nations General Assembly, 1945).

The paper also presented the authors’ GI framework, which emphasizes linking skills, values, and integrity, and connecting relationally and contributing relevantly, on behalf of the major issues facing humanity. The GI framework was used to structure the sample template (Fig. 1) in the paper. Colleagues can use and adjust this template in order to further clarify how they would like to stay informed and updated in GMH and GMS areas.

We encourage MHPs from all disciplines to get involved in GMH and for colleagues in all sectors to take advantage of the wealth of shared and synthesized GMH knowledge. Intentional and active involvement across sectors as ‘sector connectors’ is a key practice for positively impacting our precarious, often perilous, yet precious world (O'Donnell, 2013; O'Donnell and Lewis O'Donnell, 2014). The universal challenges for sustainable development and wellbeing must be met by our coordinated responses to fulfill our political and moral responsibilities, as UN Secretary-General Ban Ki-moon declares in his *synthesis report on the post-2015 sustainable development agenda*: (Ban, 2014):

Our globalized world is marked by extraordinary progress alongside unacceptable – and unsustainable – levels of want, fear, discrimination, exploitation, injustice and environmental folly at all levels…. These are universal challenges. They demand new levels of multilateral action, based on evidence and built on shared values, principles and priorities for a common destiny…. None of today's threats respect boundaries drawn by human beings, whether those boundaries are national borders or boundaries of class, ability, age, gender, geography, ethnicity or religion…. I urge Governments and people everywhere to fulfill their political and moral responsibilities. This is my call to dignity, and we must respond with all our vision and strength. (excerpts from paragraphs 11, 14, 15, 25).
